# Characterization of the E26H Mutant *Schistosoma japonicum* Glutathione S‐Transferase

**DOI:** 10.1002/prot.26794

**Published:** 2025-01-02

**Authors:** János András Mótyán, Ágota Nagyné Veres, József Tőzsér

**Affiliations:** ^1^ Laboratory of Retroviral Biochemistry, Department of Biochemistry and Molecular Biology, Faculty of Medicine University of Debrecen Debrecen Hungary

**Keywords:** affinity chromatography, fusion tag, glutathione S‐transferase, GST, protein purification, recombinant protein, *Schistosoma japonicum*

## Abstract

Glutathione‐S‐transferase, such as that of *Schistosoma japonicum* (*sj*GST) belongs to the most widely utilized fusion tags in the recombinant protein technology. The E26H mutation of *sj*GST has already been found to remarkably improve its ability for binding divalent ions, enabling its purification with immobilized metal affinity chromatography (IMAC). Nevertheless, most characteristics of this mutant remained unexplored to date. In this study, we performed a comparative analysis of the wild‐type and the E26H mutant *sj*GST by using in vitro as well as in silico approaches. We confirmed that the *sj*GST(E26H) protein exhibits significantly increased affinity for binding nickel ions as compared to the wild‐type. In addition, we proved that the *sj*GST(E26H) can be purified efficiently either with glutathione‐ or immobilized metal ion‐affinity chromatography, even in consecutive purification steps. The human retroviral‐like aspartic protease 1 (ASPRV1) conjugated with the *sj*GST(E26H) fusion tag was also successfully purified by using both of these affinity chromatographic approaches. Our studies revealed that the E26H mutant *sj*GST can be used as a versatile affinity tag because the modified protein retains the kinetic features of the wild‐type and its affinity towards glutathione, while can be purified efficiently by IMAC, as well.

## 1 Introduction

The glutathione S‐transferase (GST) is one of the most commonly used fusion tag in the recombinant protein technology, it is classified into the protein/domain group of fusion tags [1, 2, 3, 4]. The GST of *Schistosoma japonicum* (*sj*GST) belongs to the widely applied forms of this enzyme, it can be used for multiple purposes. GST is a very common affinity tag, the proteins that are fused this partner can be purified with affinity chromatography, and the immobilized proteins can be eluted from the solid phase by soluble reduced glutathione at ~10 mM concentration, under non‐denaturing conditions [1, 5]. It enables the detection of the fusion proteins via the measurement of the transferase activity of GST or by using immunoassays. GST is considered to be a versatile fusion tag, because tagging with GST can be applied in order to increase the solubility [6] or to enhance the expression of the fusion partner [7]. Due to its advantageous features, GST is frequently used in structural studies, including determination of protein structures by X‐ray crystallography, and in protein–protein interaction studies (e.g., by GST pull‐down assays) [8, 9, 10], or to prevent the proteolytic degradation of the recombinant proteins in *E. coli* cells [11].

**FIGURE 1:**
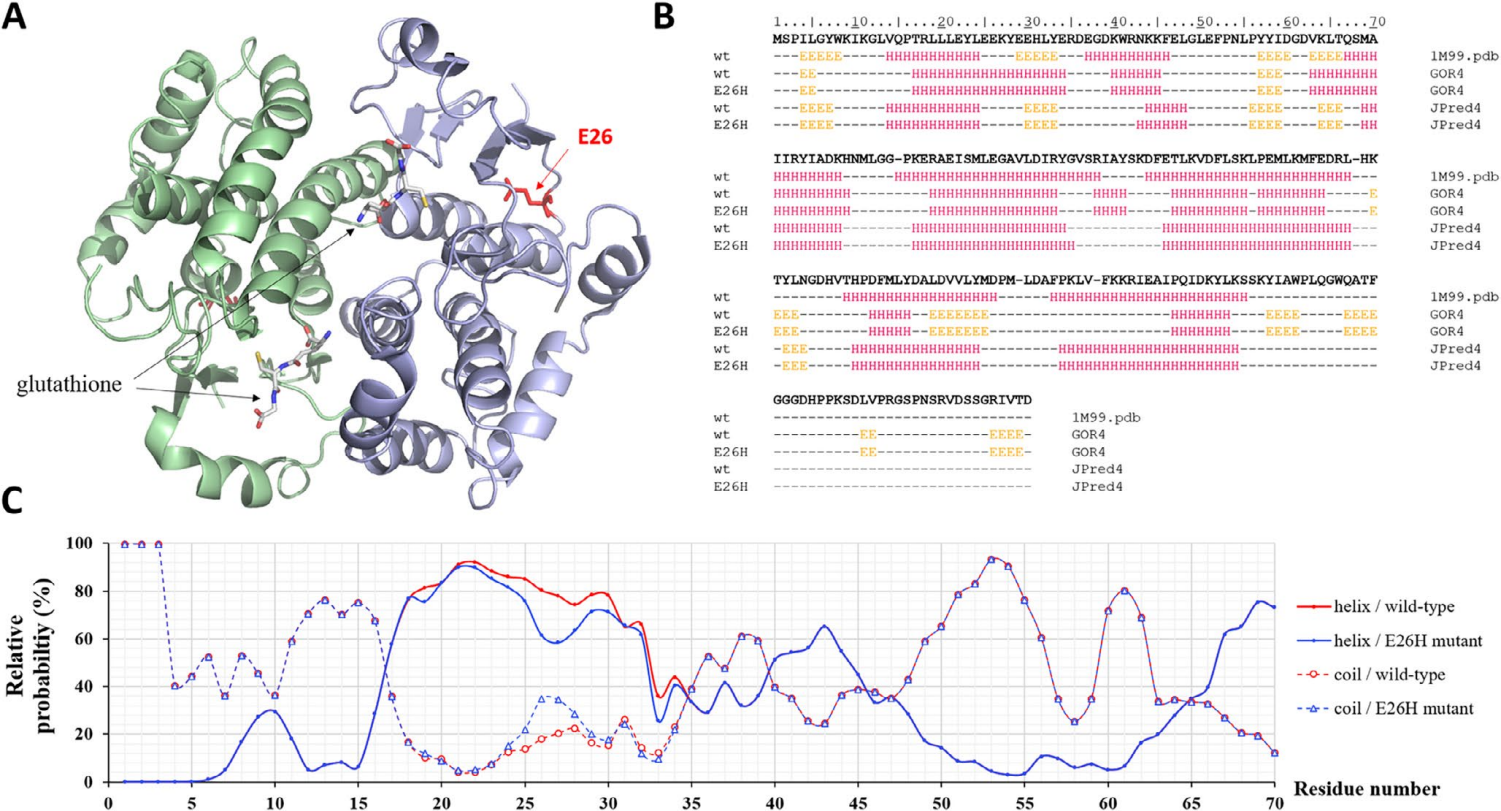
Quaternary and secondary structure of *sj*GST. (A) The overall structure of the *sj*GST homodimer is represented based on its crystal structure (5GZZ.pdb) [17]. The monomers are shown by light green and blue colors. The glutathione molecules binding to the active sites are shown by sticks, the E26 residue is red. (B) Arrangements of the secondary structural elements are shown based on the crystal structure of wild‐type (wt) *sj*GST (1M99.pdb) and based on in silico predictions (using GO4R4 and JPred4 online tools) for the wild‐type and E26H mutant proteins. “E”: strand, “H”: helix, “–”: coil. The sequence of the wild‐type *sj*GST was downloaded from the UniProt database (UniProt ID: P08515). (C) The graph represents the relative probabilities of the helices and coils (for the 1–70 region) predicted by GOR4.

A number of expression vectors coding for GST as a fusion partner are available. For example, multiple members of pGEX vectors can be used to express recombinant proteins fused to an *N*‐terminal *sj*GST, and various vectors are available that code for different protease cleavage sites for enzymatic removal of the fusion partner, such as for PreScission protease, thrombin or FXa [12, 13]. GST‐fused proteins may be expressed in *E. coli* cells using pCold‐GST plasmid system, as well [14]. The GST can be attached either to the *N*‐ or *C*‐termini of the recombinant proteins, but it can improve solubility more efficiently if positioned at the *N*‐terminus [6]. The functional GST is homodimer (see later in Figure 1A), the dimerization is the prerequisite for the formation of active sites which bind glutathione [15, 16].

The *sj*GST fusion tag is commonly utilized in recombinant protein technology, therefore, the identification of the factors that might potentially limit its application and the improvement of the efficacy of its immobilization, purification and elution is of a special importance. In addition, not only the wild‐type protein but its modified forms have also been investigated [18, 19, 20, 21, 22, 23, 24, 25].

As compared to the short epitope tags (e.g., FLAG, His_6_) [26], a possible limitation of the GST protein tag might be its relatively large size. Each subunit of the homodimer has 26 kDa molecular weight, they may interfere with the function of the fusion partner [15, 27]. The interaction between the GST fusion tag and its partner might be mediated by disulphide bonds. The formation of the unwanted interactions can be prevented by the substitution of C85, C138, and C178 surface cysteines with serines, and the modification at these sites were found to decrease the susceptibility for oxidation, as well [21, 22, 25]. However, the formation of disulphide bonds might be desired if they are formed between the subunits of a heterodimeric GST, this might reduce the formation of artificial intertwined dimers between the fusion proteins in pull‐down assays via homodimerization of GST [18]. In addition, the GST‐fused proteins can be efficiently purified only if the GST is properly folded. The influence of GST dimerization on the fusion partner may also be disadvantageous, in these cases the proteolytic removal of the GST tag is necessary to avoid changes of the fusion partner's activity.

The wild‐type *sj*GST can bind metal ions with low affinity [28], but the E26H mutation was found to provide an improved binding affinity for the protein [29]. The E26 residue does not constitute a part of the ligand‐binding site or the dimer interface (see later in Figure 1A). Rather, the E26 residue of *sj*GST is located in a position that is structurally equivalent to the D26 residue of the GST of *Clonorchis sinensis* (*cs*GST). The *cs*GST was found to have the ability for zinc binding, the binding of this ion is coordinated by the D26 and H79 residues [29, 30]. The H79 residue constitutes a part of a metal ion binding site, and in addition, it is a part of an interface that mediates protein–protein interactions [31]. This histidine is more conserved in the 79th than the glutamate in the 26th position (according to *sj*GST), GST of *Clonorchis sinensis* and *Mus musculus* contain aspartate and serine in the equivalent position, respectively [29, 31]. It was assumed that the substitution of the E26 residue with histidine might improve the ability of *sj*GST for zinc binding, the newly introduced residue was expected to contribute to the binding of the zinc ion, similar to several zinc‐binding proteins. Accordingly, the E26 residue was mutated to histidine, and—as it was expected—the modification conferred metal ion‐binding ability for *sj*GST [19]. The *sj*GST(E26H) protein was efficiently immobilized to nickel nitrilotriacetic acid (Ni‐NTA) surface and purified with immobilized metal affinity chromatography (IMAC) [29], which proved that the mutant *sj*GST can be purified due to its affinity to metal ions.

Although, it has been described that the E26H mutation increases nickel ion‐binding ability of *sj*GST, the effects of the mutation were not explored in details so far. Multiple features of the mutant enzyme, such as the effect of the mutation on glutathione binding, enzyme activity, and dimerization remained to be determined. In addition, it has not been tested whether both the metal ion‐ and glutathione‐binding abilities of *sj*GST can be used for purification by using either glutathione‐ or nickel affinity chromatography. In this work, we describe the comparative analysis of wild‐type and E26H mutant *sj*GST proteins, with a special emphasis of the binding affinities to glutathione and NTA surfaces. In addition, the modified *sj*GST has been tested for tagging and purification of a recombinant fusion protein.

## 2 Materials and Methods

### 2.1 In Silico Predictions

The sequence of *sj*GST was downloaded from UniProt database (UniProt ID: P08515). The structure of wild‐type *sj*GST was obtained from Protein Data Bank (1M99.pdb) [15]. The GOR4 (https://npsa‐prabi.ibcp.fr/cgi‐bin/npsa_automat.pl?page=npsa_gor4.html) [32], JPred4 (http://www.compbio.dundee.ac.uk/jpred/) [33], I‐Mutant 2.0 (https://folding.biofold.org/i‐mutant/i‐mutant2.0.html) [34], Site Directed Mutator (SDM) (http://marid.bioc.cam.ac.uk/sdm2/) [35], DynaMut (https://biosig.lab.uq.edu.au/dynamut/) [36], and the Metal Ion‐Binding site prediction and modeling server (MIB2) (http://combio.life.nctu.edu.tw/MIB2/) [37] online tools were applied to predict the effects of E26H mutation on the secondary structure, stability and ion‐binding affinity, respectively.

### 2.2 Expression Plasmids and Mutagenesis

The empty pGEX‐4T‐3 plasmid coding for the wild‐type *sj*GST was ordered from GE Healthcare (Chicago, IL, USA). In order to introduce E26H mutation to GST, the coding sequence in pGEX‐4T‐3 plasmid was modified by site‐directed mutagenesis using the 5′‐GGAATATCTTGAA**CAC**AAATATGAAGAGCATTTGTATGAGCGCG‐3′ oligonucleotide primer (the codon coding for the histidine residue is underlined). The mutagenesis was performed by using the QuikChange Lightning Multi Site‐Directed Mutagenesis Kit (210513; Agilent Technologies), based on the manufacturer's instructions. The success of mutagenesis was confirmed by a sequencing service (Eurofins Genomics, Germany), using the 5′‐GCACTCCCGTTCTGGATAATG‐3′ oligonucleotide primer. DNA extraction and plasmid DNA purification was performed by using High‐Speed Plasmid Mini Kit (GeneAid).

The maltose‐binding protein (MBP) containing an *N*‐terminal hexahistidine tag (His_6_‐MBP) protein was expressed from empty pDest_6_‐His‐MBP‐mApple plasmid which expression construct was in‐house stock [38, 39, 40]. The *C*‐terminus of the expressed His_6_‐MBP protein contained the cleavage site sequence of tobacco etch virus (TEV) protease (ENLYFQ*G), followed by 14 additional residues in its *C*‐terminus [38, 39, 40].

### 2.3 Protein Expression

The purified expression plasmids were transformed into BL21(DE3) competent *E. coli* cells (New England Biolabs, Ipswich, MA, USA) by heat shock. The bacteria were grown in Luria‐Bertani (LB) medium containing 0.1 w/v% ampicillin at 37°C while continuously shaking. The protein expression was induced by the addition of 1 mM isopropyl β‐d‐1‐thiogalactopyranoside (IPTG) when the optical density measured at 600 nm was between 0.6 and 0.8, followed by incubation of the suspensions at 37°C for 3 h while continuously shaking at 250 rpm. The cells then were harvested by centrifugation (5000 *g*, 20 min, 4°C). The cell pellets were suspended in phosphate buffered saline (PBS) (Sigma–Aldrich, P4417) (10 mM phosphate, 2.7 mM potassium chloride, 137 mM sodium chloride, pH 7.3) buffer containing phenylmethanesulfonyl‐fluoride (PMSF, 25 μg/mL final concentration), lysozyme (1 mg/mL final concentration) and DNAse (10 U/mL final concentration). After vortexing, the suspensions were incubated on ice for 10 min, followed by sonication on ice for 3 min (in 30 s cycles). The cell lysates were cleared by elimination of cell debris using centrifugation (10 000 *g*, 20 min, room temperature), the proteins were then purified from the supernatants.

### 2.4 Purification by Using Magnetic Affinity Beads

Glutathione High Capacity (Sigma–Aldrich, G0924) and Pierce Ni‐NTA (Thermo Scientific, #78606) magnetic agarose beads were used, by following the instructions of the manufacturers. The beads were separated and collected by using Dynamag‐2 magnetic particle concentrator (Thermo Fischer Scientific, Invitrogen).

The magnetic bead‐based experiments were performed by using 2.0 mL Protein Lobind microcentrifuge tubes (Eppendorf), the incubations were performed by shaking the beads at 750 rpm using a thermo shaker with an SC‐24 accessory block (Biosan, TS‐100).

The binding of wild‐type and E26H mutant *sj*GST proteins to nitrilotriacetic acid (NTA) and glutathione affinity beads was studied as it is described as follows.

The NTA beads were coated with different divalent ions during their regeneration by incubating them in buffers containing 100 mM CoCl_2_, CuSO_4_ or NiSO_4_, based on the protocol described previously [38, 39, 40].

For the investigation of binding affinities and for the purification, the *sj*GSTs were immobilized from cleared cell lysates or from solutions of previously purified proteins. Then, the beads were washed in consecutive steps with buffers of increasing eluent concentration while continuously shaking the tubes for 1 min. In case of Ni‐NTA and glutathione affinity beads, soluble imidazole and glutathione were used as eluents, respectively. After incubation, the supernatants were collected and subjected for electrophoresis. Each analysis was performed at least in triplicates.

### 2.5 Purification of *sj*GST by Affinity Chromatography

The cells were suspended in PBS (pH 7.4) and lysed by sonication for 3 × 30 s on ice, followed by centrifugation (4500 *g*, 15 min, 4°C). The cleared cell lysates (supernatant fraction) were filtered using 70 μm filter, then the *sj*GST proteins were purified with affinity chromatography using ÄKTA Start Protein Purification System (Cytiva).

For GST affinity purification, we used GSTrap Fast Flow (Cytiva, 17‐5130‐01) 1 mL columns. The binding buffer was PBS (pH 7.4), while 50 mM Tris–HCl buffer containing 20 mM reduced glutathione (pH 8.0) was used for isocratic elution at 1 mL/min flow rate.

For Ni‐NTA chromatography, HisTrap Fast Flow (Cytiva, 17‐5319‐01) 1 mL columns were applied. PBS (pH 7.4) was used for binding and equilibration, and 50 mM Na‐phosphate buffer containing 150 mM NaCl and 200 mM imidazole (pH 7.5) was used for linear gradient elution at 1 mL/min flow rate.

Thermo Scientific Slide‐A‐Lyzer Dialysis Cassettes (MWCO: 10K, Cat. no: 66380) were utilized to dialyze the purified fractions against PBS (pH 7.4) at 4°C while continuously stirring the solution.

### 2.6 Electrophoresis and Western‐Blot

The sodium dodecyl sulphate (SDS) polyacrylamide gel electrophoresis (PAGE) was performed by using 12% or 15% polyacrylamide gels. Denaturing conditions were applied for sample preparation as well as electrophoresis. The samples to be analyzed were supplemented with loading dye containing sodium dodecyl sulphate (SDS) and β‐mercaptoethanol, then, were incubated at 95°C for 10 min. After electrophoresis at constant 110 V voltage, the gels were stained by Coomassie Brilliant Blue dye. The band intensities were quantified by using GelAnalyzer 2010a freeware software (www.gelanalyzer.com; István Lázár Jr., PhD and István Lázár Sr., PhD, CSc).

If the proteins were separated at non‐denaturing conditions, precast native gel (4%–15%, #4561084, Bio‐Rad) and Tris‐/glycine running buffer (#1610734, Bio‐Rad), native sample loading dye (#1610738, Bio‐Rad) and NativeMark unstained protein standard (LC0725, ThermoFischer Scientific) were applied.

The Western‐blot was performed as described previously [41]. After blocking the membrane with 5% dry milk in Tris‐buffered saline buffer (TBS, pH 7.5) overnight at 4°C, the polyclonal anti‐GST antibody (27457701 V, Cytiva) was applied in 5000X dilution (in TBS complemented with Tween20 (TTBS) and containing 5% dry milk). Anti‐goat IgG was used as secondary antibody in 20 000X dilution (in TTBS containing 5% dry milk). The proteins were detected using Advansta K‐12045‐D50 WesternBright ECL Western blot detection kit. Gel and blot imaging was performed by using AZURE 600 Imaging System (Azure Biosystems).

### 2.7 Enzyme Activity Measurements

The enzyme activity measurements were performed by using 1‐chloro‐2,4‐dinitrobenzene (CDNB; Sigma–Aldrich, #138630) as substrate, using the conditions recommended by the manufacturer. The reactions were performed in 100 mM potassium phosphate buffer containing 1.0 mM ethylenediaminetetraacetic acid (EDTA) (reaction buffer, pH 6.5) and the mixtures were incubated at 25°C for 10 min. The reaction mixtures (300 μL final volume) were prepared in the wells of 96‐well plates and contained 270 μL reaction buffer, 10 μL reduced glutathione (75 mM in reaction buffer), 10 μL substrate (7.5–300 mM in reaction buffer), and 10 μL enzyme (0.036 mg/mL in PBS). The change of absorbance was measured continuously at 340 nm wavelength using Synergy H1 plate reader. Statistical analysis (unpaired t test) was performed by using T test calculator online tool of GraphPad (https://www.graphpad.com/quickcalcs/ttest1/; accessed at 17.02.2023).

### 2.8 Expression and Purification of *sj*GST(E26H)‐ASPRV1‐28(D212A) Protein

The pGEX‐4T‐3 plasmid coding for the wild‐type ASPRV1‐28 was in‐house stock [42]. The E26H mutation was introduced into the *sj*GST based on the protocol described in the *Expression plasmids and mutagenesis* section. The D212A mutation was introduced into the coding sequence of ASPRV1‐28 by the QuikChange Lightning Multi Site‐Directed Mutagenesis Kit (210513; Agilent Technologies), based on the manufacturer's instructions and using the 5′‐GTGAGGTTCCTGGTGGCCTCTGGGGCCCAGGTC‐3′ mutagenesis primer.

The *sj*GST(E26H)‐ASPRV1‐28(D212A) fusion protein was expressed from the pGEX‐4T‐3 expression plasmid in *Escherichia coli* BL21(DE3) cells, using the expression conditions described previously for the *sj*GST‐ASPRV1‐28(wild‐type) protein [42]. The recombinant *sj*GST(E26H)‐ASPRV1‐28(D212A) was extracted from inclusion bodies based on the protocols we used previously for the wild‐type ASPRV1‐28 [42]. For purification, we used a 50 mM MES and 300 mM NaCl‐containing buffer (pH 6.0) described by Han et al. [29], which was supplemented with 0.005 M EDTA and 2% sarcosyl for cell lysis, based on the protocol of Golda et al. [42]. First, purification was carried out with Ni‐NTA affinity chromatography by using HisTrap Fast Flow column. After the gradient elution of the immobilized proteins (0%–100% linear gradient, using phosphate buffer containing 400 mM imidazole), the eluted fractions were further purified with glutathione affinity chromatography by GSTrap Fast Flow column (using Tris–HCl buffer containing 20 mM reduced glutathione for isocratic elution). Chromatographic separation was performed by using ÄKTA Start Protein Purification System (Cytiva), the proteins were analyzed by SDS‐PAGE using 15% polyacrylamide gel, and gel imaging was performed by using AZURE 600 Imaging System (Azure Biosystems).

## 3 Results

### 3.1 In Silico Analysis of the Effects of E26H Mutation

To predict possible effects of E26H mutation on the protein structure and stability, sequence‐ and structure‐based in silico approaches were applied (Table 1). The E26 residue is located in a loop, next to the *C*‐terminus of an α‐helix (based on its crystal structure; PDB ID: 3ISO) [29]. The GOR4 and Jpred4 secondary structure prediction algorithms implied that the 26th residue is located within a helical and a coiled region, respectively. None of the applied algorithms predicted the rearrangement of secondary structural elements (e.g., from helix to coil or to strand) at the site or in the proximity of the modified position upon the E26H mutation (Figure 1B,C). The secondary structural organizations that were predicted by the computational analyses and determined based on a crystal structure of *sj*GST (1M99.pdb) [15] were in good agreement (Figure 1B); accordingly, the results of the predictions were estimated to be sufficiently reliable.

**TABLE 1:** Effect of E26H mutation on *sj*GST's secondary structure and stability. The sequence of *sj*GST was used for predictions, while the structure‐based analyses were performed based on a crystal structure of wild‐type *sj*GST (1M99.pdb). The result of secondary structure prediction is represented in Figure 1B.

Prediction	Secondary structure		Stability		
	GOR4	JPred4	I‐Mutant 2.0	SDM	DynaMut
Sequence‐based	No change	No change	Destabilizing (−0.31 kcal/mol)	—	—
Structure‐based	—	—	Destabilizing (−1.33 kcal/mol)	Stabilizing (0.13 kcal/mol)	Stabilizing (0.928 kcal/mol)

The potential changes of the protein stability upon the point mutation were predicted by using both sequence‐ and structure‐based algorithms (Table 1). The I‐Mutant2.0 web server [23] can be used to predict the changes of protein stability (DDG, kcal/mol) upon single point mutations, either from protein structure or sequence. The Site Directed Mutator (SDM) [35] calculates stability score for the point mutations that reflects the free energy differences between the wild‐type and mutant proteins. The DynaMut algorithm [36] can be used to analyze and visualize protein dynamics and predict the effects of mutations on protein dynamics and stability that are caused by vibrational entropy changes.

The I‐Mutant 2.0 program was the only one which predicted the E26H mutation to be destabilizing, while the SDM and DynaMut algorithms implied stabilizing nature for the mutation. The results of secondary structure predictions and stability analyses also implied that the E26H mutation is rather neutral or has only moderate effect on the overall stability, indicating the *sj*GST most probably retains its structural integrity upon the mutation.

The effect of the E26H mutation was analyzed by the Metal Ion‐Binding site prediction and modeling server (MIB2), as well [37]. This computational approach uses the AlphaFold protein structure database to acquire 3D information for metal ion docking and binding site analysis. We used the structural coordinates of the wild‐type and the mutant *sj*GST in order to identify potential binding sites of Cu^2+^, Co^2+^, and Ni^2+^ divalent ions as well as to calculate binding probability scores for these metal ions (Table 2).

**TABLE 2:** Effect of E26H mutation on *sj*GST's predicted ion‐binding ability. The structure‐based analyses were performed based on the structure of wild‐type *sj*GST (1M99.pdb) and based on the structure of E26H mutant that was predicted by DynaMut web server based on 1M99.pdb. The predicted ion‐binding residues and scores are shown in the table.

	Enzyme	Cu^2+^	Ni^2+^	Co^2+^
Predicted score	Wild‐type	3.776	0.419	1.161
	E26H	4.201	3.263	3.230
Number of binding sites containing the 26th residue	Wild‐type	1	0	0
	E26H	9	2	2
Predicted residues of the most probable site containing the 26th residue	E26H	L21, L24, H26, H79	E22, H26	E22, H26

The MIB2 predictions implied that the wild‐type *sj*GST can potentially bind Cu^2+^ ions efficiently, while the predicted binding probabilities were remarkably lower for Co^2+^ and Ni^2+^ ions. The E26H mutation was predicted to improve divalent ion‐binding ability of *sj*GST and increases the number of the possible binding sites, in the case of each studied ion. The binding probability showed only ~10% increase for the Cu^2+^ ions, while there was a 3‐ and 8‐fold increase in the case of Co^2+^ and Ni^2+^ ions, respectively. The slight difference predicted for the Cu^2+^ ion implied that the wild‐type and mutant proteins might not exhibit differences in the binding to Cu‐NTA surface.

### 3.2 Binding of *sj*GST to Different Divalent Ions

The binding affinities of the wild‐type and mutant *sj*GST proteins to different divalent ions were investigated experimentally. After immobilizing the proteins to NTA magnetic beads, the affinity surfaces were washed in consecutive steps with buffers of increasing eluent (imidazole) concentration, and the collected fractions were analyzed by SDS‐PAGE.

**FIGURE 2:**
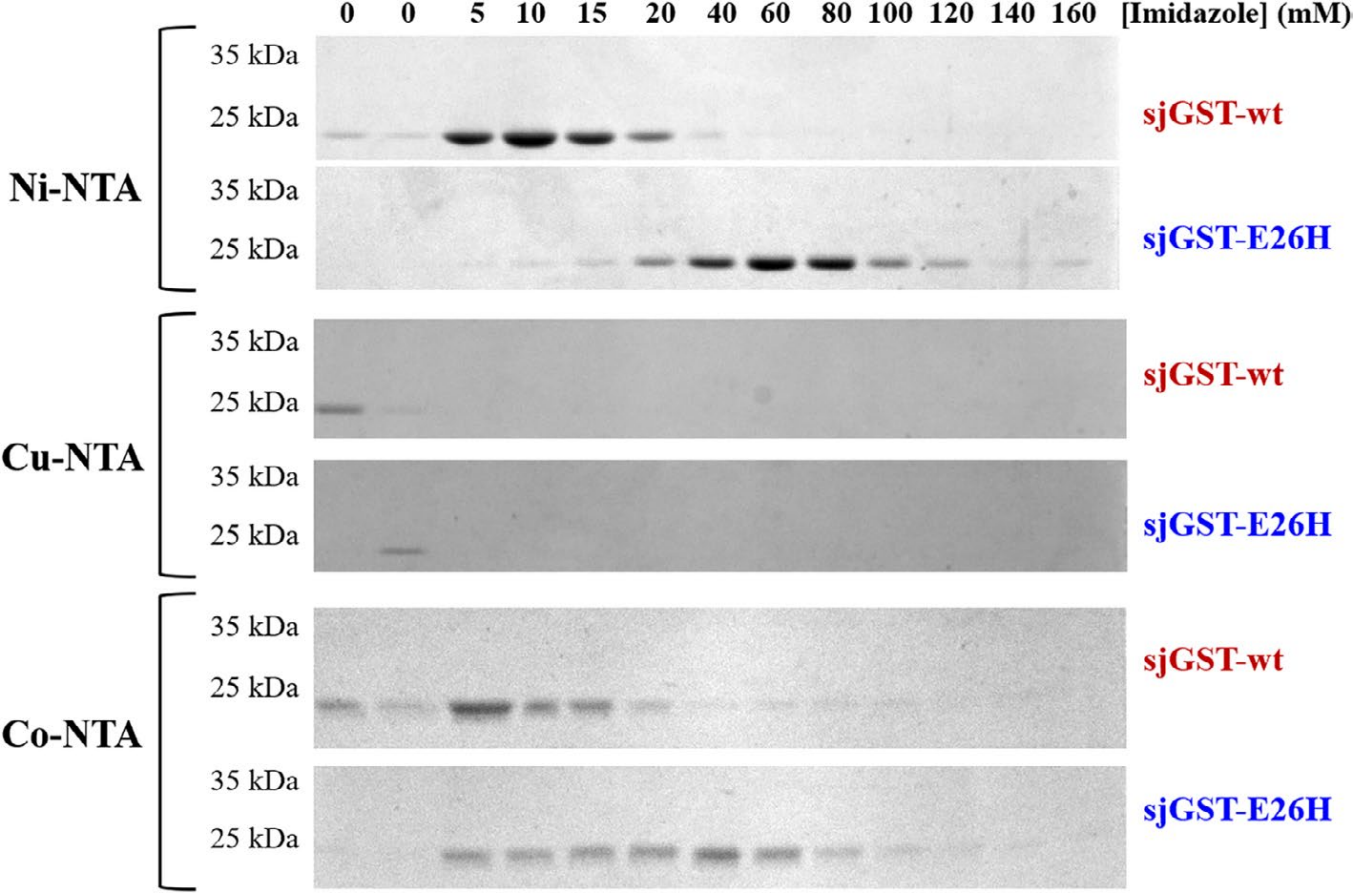
Binding of wild‐type and E26H mutant *sj*GSTs to Ni‐, Cu‐, and Co‐NTA surfaces. The beads were washed in consecutive steps with buffers of increasing eluent (imidazole) concentration, the collected fractions were analyzed by SDS‐PAGE. Representative gel images are shown based on the results of three independent experiments.

Considerably higher binding affinity was observed for the E26H mutant to the Ni‐NTA surface as compared to that of the wild‐type (Figure 2). This was in agreement with the in silico predicted existence of a Ni^2+^ binding site in the mutant protein which was absent from the wild‐type. The MIB2 prediction implied Cu^2+^ binding ability for both wild‐type and E26H mutant *sj*GSTs and higher number of putative binding sites in the mutant, but none of the enzymes exhibited sufficient binding to the Cu‐NTA beads. We observed elevated binding of the E26H mutant to Co‐NTA surface as compared to the wild‐type (Figure 2). This is in agreement with the predictions which implied that the E26H mutation of *sj*GST confers its ability for binding Co^2+^ ion, while binding sites for this metal ion—that encompass the E26 residue—are absent from the wild‐type (Table 2).

The binding probabilities predicted for the different ions were in agreement with the binding abilities determined in vitro for Ni‐ and Co‐NTA. The experimental assays implied slightly higher affinity of the E26H mutant for Ni^2+^ than for the ions Co^2+^ ions, therefore, the downstream analyses were performed by using Ni‐NTA surfaces.

### 3.3 Binding of *sj*GST to Glutathione Affinity Surface

It has not been described previously whether the E26H mutation interferes with the ability of *sj*GST for binding glutathione [29]. Therefore, we intended to investigate the binding of the wild‐type and mutant proteins to magnetic glutathione agarose beads in vitro.

**FIGURE 3:**
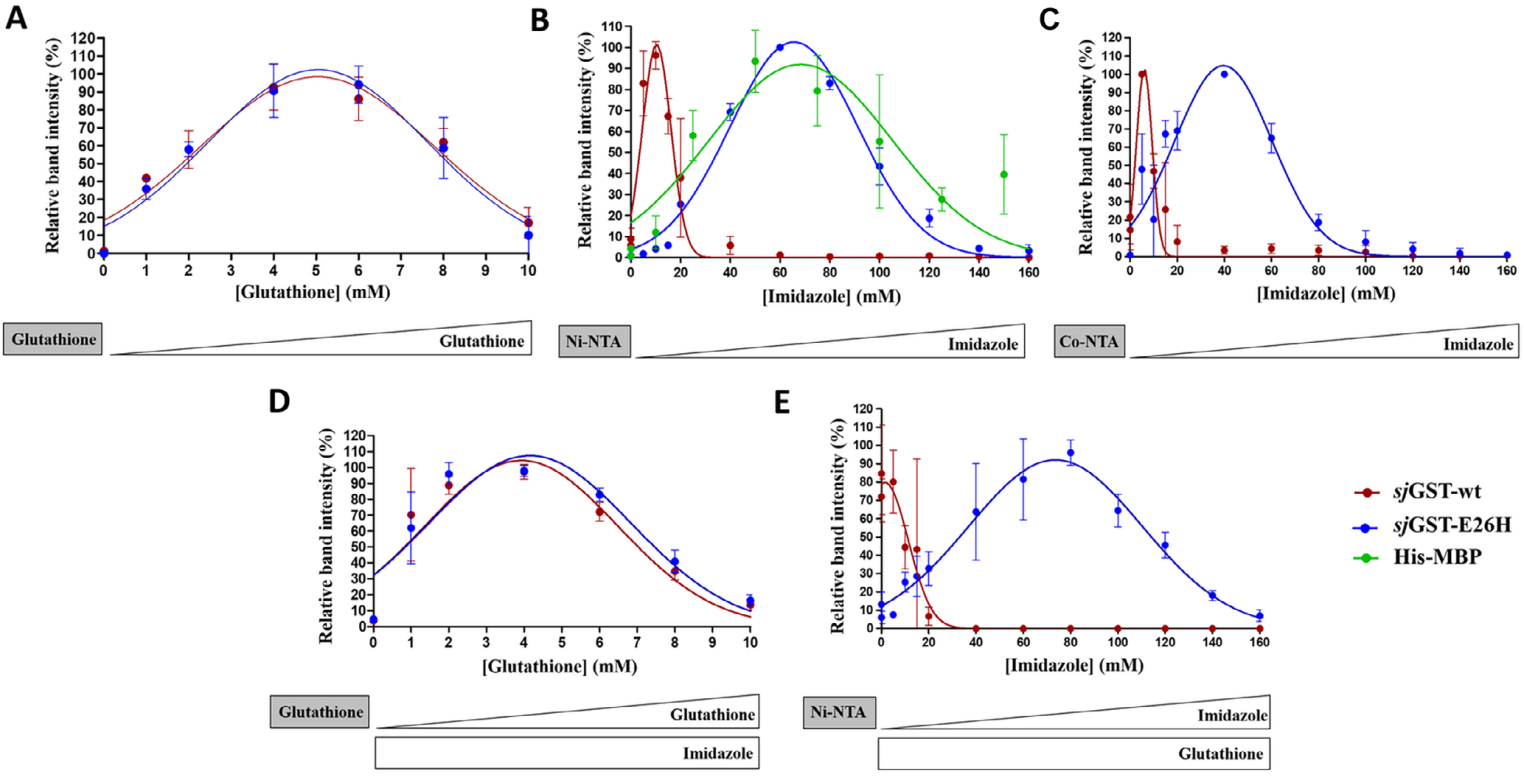
Binding of wild‐type and E26H mutant *sj*GSTs to affinity surfaces. (A) The beads were washed in consecutive steps with buffers of increasing eluent (imidazole) concentration, the collected fractions were analyzed by SDS‐PAGE. Representative gel images are shown based on the results of three independent experiments. (B) The Ni‐NTA beads were washed in consecutive steps with buffers of increasing eluent (imidazole) concentration, the collected fractions were analyzed by SDS‐PAGE. Representative gel images are shown based on the results of three independent experiments. (C) The Co‐NTA beads were washed in consecutive steps with buffers of increasing eluent (imidazole) concentration, the collected fractions were analyzed by SDS‐PAGE. Error bars represent SD (*n* = 3). Representative gel images are shown based on the results of three independent experiments. (D) The effect of glutathione on the binding of *sj*GST to glutathione affinity surface. The binding affinities of wild‐type and E26H mutant *sj*GSTs to glutathione affinity surface were studied in the presence of soluble imidazole (200 mM final concentration). (E) The binding affinities of wild‐type and E26H mutant *sj*GSTs to Ni‐NTA affinity surface were studied in the presence of soluble glutathione (10 mM final concentration). The applied affinity beads are indicated in boxes of gray background, the increasing and constant eluent (glutathione and/or imidazole) concentrations are represented schematically below the graphs by white triangles and boxes, respectively. Error bars represent SD (*n* = 3). Representative gel images are shown in Figure S1.

The wild‐type and E26H mutant proteins exhibited indistinguishable binding affinity for glutathione, the highest degree of elution was observed at 5.03 ± 0.15 and 5.04 ± 0.15 mM glutathione concentration, respectively (Figure 3A). This clearly implies that the glutathione binding is unaffected by the E26H mutation, and the modified protein can be purified with glutathione affinity chromatography as efficiently as the wild‐type form. The majority of the immobilized proteins eluted at 5 mM glutathione concentration, the residual amounts were eluted only at higher eluent concentration. The 5 mM is half of the glutathione concentration generally recommended for the elution of GST‐tagged proteins [1, 3].

### 3.4 Binding of *sj*GST to Ni‐NTA Affinity Surface

The binding affinities of the *sj*GST proteins to Ni‐NTA affinity surface were compared by determining the highest imidazole eluent concentration causing the highest degree of protein elution from the magnetic beads.

The majority of the immobilized wild‐type and the mutant proteins eluted from the beads at 10.40 ± 0.38 and 65.58 ± 0.76 mM imidazole concentrations, respectively. The mutant protein exhibited at least 5‐fold higher binding affinity as compared to the wild‐type (Figure 3B). This concentration is within the concentration range that is generally applied to elute His‐tagged proteins (20–250 mM) [1].

Our results confirmed the findings of Han et al., who described weak binding ability for the wild‐type *sj*GST and improved Ni‐binding affinity for the E26H mutant [29].

To compare the affinity of the *sj*GST(E26H) to that of a His‐tagged protein, the binding of a His_6_‐MBP protein (expressed from empty pDest‐His_6_‐MBP‐mApple plasmid) to Ni‐NTA affinity beads was also studied. The *sj*GST(E26H) and His_6_‐MBP exhibited highly similar binding affinities, the highest elution was observed at 65.58 ± 0.76 and 68.31 ± 3.35 mM imidazole concentration, respectively (Figure 3B). The imidazole concentration that caused the highest degree of elution was slightly higher for His_6_‐MBP, indicating that the affinity of the E26H mutant *sj*GST to Ni‐NTA surface resembles that of the His_6_‐MBP, at least under the applied experimental conditions and in the context of the standalone *sj*GST rather than a GST‐fused recombinant protein.

### 3.5 Binding of *sj*GST to Co‐NTA Affinity Surface

In agreement with the prediction of divalent ion‐binding residues and sites (Table 2), we observed more efficient binding for the E26H mutant to Co‐NTA surface than for the wild‐type *sj*GST (Figure 3C), therefore, we determined the binding affinities of the enzymes. The highest dissociation was observed at 6.04 ± 0.26 and 39.61 ± 1.49 mM imidazole concentration for the wild‐type and the mutant, respectively, indicating that the Co^2+^‐binding affinity of the E26H mutant is higher than that of the wild‐type.

The difference between the binding affinity of the wild‐type and the mutant *sj*GST was almost identical in the case of Ni‐ and Co‐NTA, the mutant exhibited approximately 6‐fold higher affinity for Ni^2+^ and Co^2+^ ions, as well. The overall affinity was slightly lower for Co^2+^ than for Ni^2+^ ions, therefore, the use of Ni‐NTA might be more efficient for the purification the E26H mutant with IMAC. In addition, Ni^2+^ is the most common divalent ion that is applied for IMAC.

### 3.6 Binding of *sj*GST to Affinity Surfaces in the Presence of Eluents

We tested the effects of soluble imidazole and glutathione on the immobilization of the proteins to glutathione and Ni‐NTA surfaces, respectively. This was considered to be potentially important if the E26H mutant is purified in two consecutive steps without removing the eluent after the first purification.

The Ni‐NTA or glutathione beads were washed in consecutive steps with buffers of increasing eluent concentration (imidazole or glutathione or, respectively), in the presence of respective soluble glutathione (10 mM) or imidazole (200 mM). The collected fractions were analyzed by SDS‐PAGE (Figure 3D). The binding of the wild‐type and E26H mutant proteins to the glutathione affinity surface were highly similar in the presence of imidazole (mean: 3.92 ± 0.16 and 4.15 ± 0.16 mM for the wild‐type and mutant, respectively). These values were comparable with those we obtained in the absence of imidazole (Figure 3A). The highest dissociation was observed at ~20% lower concentration of soluble glutathione eluent as compared to that when imidazole was not present (Figure 3D). The presence of soluble glutathione decreased the binding affinity of the wild‐type *sj*GST to the Ni‐NTA surface (mean imidazole concentration causing the highest elution dropped from 10.40 ± 0.37 to 1.48 ± 2.63 mM). Such a remarkable change was not observed for the E26H mutant. In the presence of glutathione, the affinity of E26H mutant for Ni‐NTA (mean imidazole concentration causing th highest elution: 73.61 ± 1.78 mM) closely resembled the affinity we observed in its absence (mean imidazole concentration: 65.58 ± 0.76 mM) (Figure 3E).

### 3.7 Enzyme Kinetic Parameters of *sj*GST

The GST‐fused proteins can be detected by measuring the transferase activity of GST [13], thus, we determined whether the modification of *sj*GST interferes with its catalytic efficiency. The ability of the *sj*GST for the binding to glutathione affinity surface was not changed by the point mutation, consequently, we expected similar catalytic properties for the wild‐type and mutant proteins. The enzyme kinetic analysis was performed by using 1‐chloro‐2,4‐dinitrobenzene (CDNB), which is commonly used in GST activity assays as substrate [11, 13, 20, 22, 23, 24, 25, 28].

**FIGURE 4:**
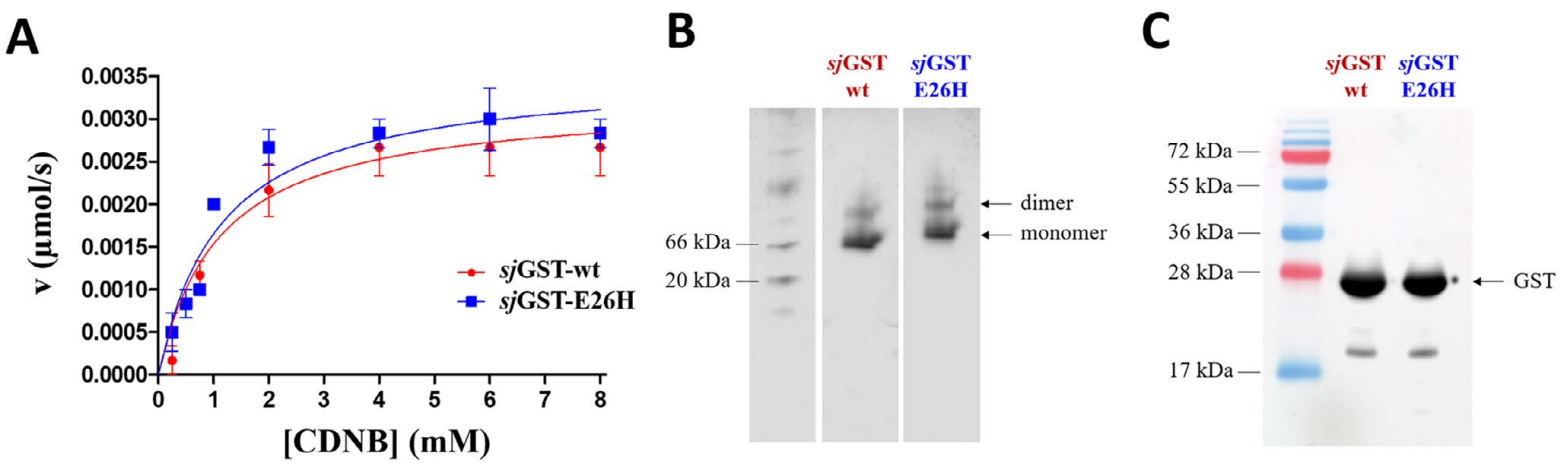
Effect of E26H mutation of *sj*GST on the kinetic parameters, dimerization and detection by Western‐blot. (A) The activity measurements were performed by using CDNB colorimetric substrate. Kinetic parameters were determined by fitting the data to the Michaelis–Menten equation, the calculated kinetic values are shown in Table 3. Error bars represent SD (*n* = 6). (B) Separation of the monomeric and dimeric *sj*GSTs is shown in a representative Coomassie‐stained native polyacrylamide gel. The figure shows different parts of the same gel, separated by white spaces. (C) Detection of purified wild‐type and E26H mutant *sj*GSTs by Western‐blot using a polyclonal anti‐GST antibody, a representative blot image is shown.

The kinetic measurements revealed highly comparable characteristics for the wild‐type and E26H mutant *sj*GSTs (Figure 4A), the catalytic constants were almost identical, the difference between the *k*_cat_/*K*_M_ values was not significant statistically (*p* = 0.2484) (Table 3). The E26H mutation does not cause change of the glutathione S‐transferase activity, because the modified residue is located far from the active site as well as the mutation does not have such distant effects which might interfere with the enzymatic catalysis.

**TABLE 3:** The wild‐type and E26H mutant *sj*GSTs have highly comparable catalytic efficiency. The kinetic curves that were used for the determination of the values are shown in Figure 4a.

	*k*_cat_ (s^−1^) (× 10^−3^)	*K*_M_ (mM)	*k*_cat_/*K*_M_ (mM^−1^ s^−1^) (× 10^−3^)
Wild‐type	74.38 ± 6.22	1.11 ± 0.29	67.25 ± 6.02
E26H	81.88 ± 5.34	1.15 ± 0.24	71.14 ± 4.92

### 3.8 Dimerization of *sj*GST

It is known that GST forms homodimers in solution; therefore, we studied whether the E26H mutation affects the dimer formation. For this purpose, the sample preparation and electrophoresis were carried out using non‐denaturing conditions (Figure 4D). The E26H mutant was detected at higher approximate molecular weight than the wild‐type, indicating its relatively slower migration under non‐denaturing conditions due to the substitution of the negatively charged surface glutamate residue to histidine. Both monomeric and dimeric enzyme forms were detected in case of the wild‐type and the mutant, as well, proving that *sj*GST retains its ability for dimerization upon the E26H mutation, as it was expected based on the results of the kinetic analysis (Table 3). The dimer formation is required for the transferase activity [15, 16], accordingly, the highly comparable kinetic parameters also imply that the wild‐type and mutant proteins do not exhibit different abilities for dimerization.

### 3.9 Detection of *sj*GST by Western‐Blot

The GST‐conjugated proteins are often detected by Western‐blot using an anti‐GST antibody. Therefore, we used a polyclonal antibody for the detection of the wild‐type and mutant proteins and found that the E26H mutant can be identified by Western‐blot as efficiently as the wild‐type (Figure 4C), proving that the modification does not decrease the efficacy of the antibody‐based detection.

### 3.10 Purification of *sj*GST Using Two Affinity Steps

**FIGURE 5:**
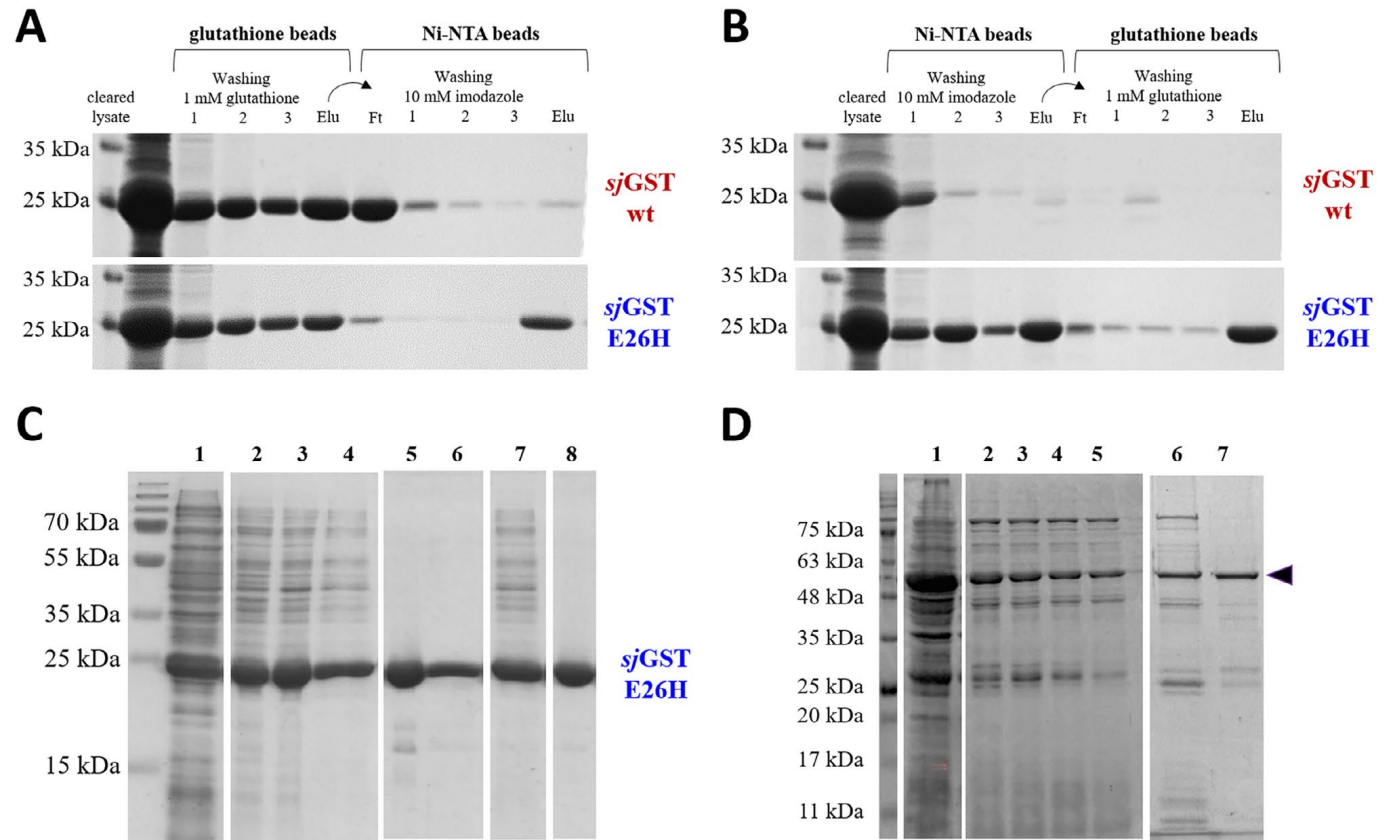
Ni‐NTA and glutathione affinity purification of *sj*GST proteins. (A) Purification of wild‐type and E26H mutant *sj*GSTs from cleared cell lysates with glutathione affinity beads, followed by IMAC. (B) Purification of wild‐type and E26H mutant *sj*GSTs with IMAC, followed by glutathione affinity beads. The eluates from the first purification steps (containing the corresponding eluent) were directly transferred to the second affinity surface, independently from the order of the two applied methods (A, B). Representative gel images show SDS‐PAGE analysis of cleared lysates, samples from washing steps (1–3), the flowthrough (Ft) and eluate (Elu) fractions. The GST‐fused proteins were present in the fractions after washing steps, as they were present in the cleared lysates in excessive amount. (C) Purification of E26H *sj*GST using two affinity chromatography steps. A representative SDS‐PAGE gel image is shown. The proteins were purified with chromatography using affinity columns, the samples are numbered as it follows: Sample 1, total cell lysate; sample 2–4, purified *sj*GST(E26H), eluate fractions from Ni‐NTA affinity chromatography; sample 5–6, purified *sj*GST(E26H), eluate fractions from glutathione affinity chromatography; sample 7, pooled 2–4. eluate fractions; sample 8, purified *sj*GST(E26H), purified in two steps (the pooled eluate fractions from Ni‐NTA purification (sample 7) were further purified with glutathione affinity chromatography). The figure shows different parts of the same gel, separated by white spaces. (D) SDS‐PAGE analysis of the samples from the expression and purification of *sj*GST(E26H)‐ASPRV1‐28(D212A). Sample 1. Total cell lysate; Sample 2–5. Eluate fractions from the purification of *sj*GST(E26H)‐ASPRV1‐28(D212A) with Ni‐NTA affinity chromatography (in total 25 × 1 mL fractions were collected during the linear gradient elution (from 0 to 400 mM imidazole), eluate fraction 14–17 are represented); Sample 6. Pooled eluate fractions from Ni‐NTA purification (2–5) that were subjected for glutathione affinity chromatography; Sample 7. Eluate fraction of *sj*GST(E26H)‐ASPRV1‐28(D212A) from glutathione affinity chromatography. The figure shows different parts of the same gel, separated by white spaces. The arrowhead indicates the GST‐fused ASPRV1‐28 protein.

We found that neither imidazole nor glutathione interfere with the binding of *sj*GST to respective glutathione and metal ion affinity surfaces (Figure 3). Therefore, we assumed that the E26H mutant protein might be purified in two consecutive affinity steps. Accordingly, the proteins were immobilized to the Ni‐NTA beads, the beads were then washed with buffer containing imidazole in low concentration, followed by elution using imidazole. The eluent fraction was then directly transferred to glutathione affinity surface and purified by using soluble glutathione for elution. The protocol was performed by using the glutathione affinity purification in the first and the Ni‐NTA in the second step, as well. The fractions were analyzed by SDS‐PAGE (Figure 5).

As expected, the wild‐type *sj*GST was purified efficiently only with glutathione affinity beads, it was not detected in the eluate fraction using Ni‐NTA for purification either in the first or the second step (Figure 5A,B). In contrast to this, the E26H mutant was successfully purified even using glutathione or metal ion affinity surface in the first purification step (Figure 5A,B). This implied that if necessary, the mutant *sj*GST can be further purified without removing the eluent before a second purification step, which makes the application of buffer exchange (e.g., by ultrafiltration or dialysis) unnecessary and improves the time‐ and cost‐efficiency of the purification protocol.

The purification of *sj*GST(E26H) was performed not only by using magnetic affinity beads but also by using glutathione and Ni‐NTA affinity columns, as well, which resulted in efficient purification of the mutant (Figure 5C). The eluent fractions from a single Ni‐NTA purification (sample 2–4 in Figure 5C) contained considerable amount of contaminants at the higher molecular weight range, which were present in remarkably lower amount if a single glutathione affinity purification was performed (sample 5–6 in Figure 5C). Further purification of the pooled Ni‐NTA eluates (sample 7 in Figure 5C) with glutathione affinity chromatography improved the purity of the eluate, the purity increased from 67% to 95% (sample 8 in Figure 5C). In the parallel experiments, the purity of the E26H mutant was similar if it was purified in a single (glutathione affinity purification) or in two affinity purification steps (Ni‐NTA followed by glutathione affinity purification). Although, the E26H mutant can be purified with Ni‐NTA, based on our experiences a second affinity step may be required if the purity is not sufficiently high. It is important to note that not only high purity but high yield may also be desired, this needs to be considered while designing a sufficient purification strategy.

### 3.11 Expression and Purification of a GST‐Tagged Protein

In order to apply the *sj*GST(E26H) fusion tag for the purification of a GST‐tagged protein, we have chosen a human protein, the retroviral‐like aspartic protease 1 (ASPRV1) [42, 43, 44, 45, 46]. ASPRV1 is expressed as a 37 kDa protein (ASPRV1‐37) which contains a retrovirus gag‐like domain (being similar to the retrovirus capsid protein) close to its *N*‐terminus and a *C*‐terminal retroviral protease‐like domain. The full‐length ASPRV1‐37 precursor undergoes auto‐proteolysis which releases a 28 kDa protein (ASPRV1‐28) whose additional self‐cleavages release the 14 kDa protease domain (ASPRV1‐14) [42, 43, 46]. We have already studied the recombinant ASPRV1‐28 and ASPRV1‐14 proteins, but both protein forms have been fused to the wild‐type *sj*GST [42].

In this work, the primarily designed expression construct [42] was modified in order to introduce the E26H mutation to *sj*GST as well as the D212A mutation to ASPRV1‐28. The D212 residues corresponds to the catalytic aspartate of the conserved D‐S/T‐G‐A active site motif of the protease [44]. The substitution of the aspartate with alanine was expected to inactivate the protease and prevent the release of the GST tag and ASPRV1‐14 from the ASPRV1‐28 precursor, because previously we have observed the release of GST tag via autoproteolysis even during the expression and purification of the fusion protein [42]. The *sj*GST(E26H)‐ASPRV1‐28(D212A) protein was expressed in a bacterial expression system, using the protocols described previously [42].

The *sj*GST(E26H)‐ASPRV1‐28(D212A) protein was purified first with Ni‐NTA chromatography at high salt concentration (300 mM NaCl), based on the protocols described previously [29]. The recombinant protein was successfully immobilized onto Ni‐NTA surface, and significant amount of contaminants were removed by this purification step (Figure 5d). In agreement with the results of Han et al. [29], the recombinant protein eluted from the column at a relatively high imidazole concentration. It is important to note that a purification performed at lower salt concentration (by using 50 mM Na‐phosphate buffer containing 150 mM NaCl, pH 7.5) resulted only in insufficient purity; the efficacy of purification was higher if buffer of high salt concentration was applied. In order to improve the overall purity of the recombinant protein, after Ni‐NTA purification, pooled eluate fractions of *sj*GST(E26H)‐ASPRV1‐28(D212A) were further purified with glutathione affinity chromatography. As expected, a second affinity purification improved the purity and contaminants were detected in the eluate fractions in remarkably lower amount (Figure 5D).

In this study, we successfully applied Ni‐NTA for the purification of both the free *sj*GST(E26H) (Figure 5C) and the GST‐tagged ASPRV1‐28(D212A) fusion protein (Figure 5D). Our results are in agreement with those of Han et al., who purified the human biotin protein ligase (hBPL) fused to *sj*GST(E26H) with IMAC and obtained sufficient purity [29]. After the Ni‐NTA purification, we subjected the eluate fractions for an additional purification with glutathione affinity chromatography, which improved the purity of the Ni‐NTA‐purified protein.

## 4 Discussion

The *sj*GST is widely applied in the recombinant protein technology due to its versatile features, nevertheless, it is a special interest to identify sequence variants and mutants with improved characteristics. In accordance with this, we aimed to investigate the E26H mutant *sj*GST fusion tag protein, which has been reported earlier in the scientific literature to exhibit higher affinity towards divalent Ni^2+^ ions as compared to the wild‐type and thus can be purified with Ni‐NTA [29], nevertheless, the effects of the E26H mutation remained unclear. Therefore, we performed a comparative analysis of the wild‐type and E26H mutant *sj*GSTs by using in silico as well as in vitro approaches.

First, in silico approaches were utilized to estimate possible effects of the E26H mutation on the secondary structure, stability as well as metal ion binding ability of *sj*GST. The predictions revealed that the enzyme retains its structural integrity upon the mutation, changes of the secondary and the tertiary structure as well as the overall stability were not predicted. An in silico metal ion‐binding site prediction analysis (using MIB2 web server) implied improved binding of Ni^2+^ ions to *sj*GST(E26H). The 26th residue is not part of the dimerization surface [16], thus, the mutation of *sj*GST in this position was not expected to have any direct effect on the interactions between the monomers. The E26 residue is not involved in ligand binding, but the in silico analyses were unable to predict any distant effects which may potentially change catalytic properties. To prove the results of computational predictions, we determined experimentally the affinities towards different divalent ions as well as the abilities for dimerization and enzymatic catalysis, followed by the comparison of the in vitro and in silico data.

A possible limitation of this study might be that the structural characteristics of the E26H mutant protein were not investigated in details experimentally (e.g., by using circular dichroism (CD) or fluorescent spectroscopy) or by using extended molecular dynamic calculations. Although, such analyses might reveal the structural background of metal ion binding at atomic level, the primary aim of this work was to study the mutant *sj*GST from the viewpoint of its practical application and its purification with IMAC and/or with glutathione affinity chromatography.

We performed multiple binding assays which confirmed that the E26H mutation improves binding of *sj*GST to Ni‐NTA surface [29] and proved that the mutation does not interfere with ability for binding glutathione. The E26H mutant protein showed efficient binding to Co‐NTA surface, as well, and the binding to Ni‐NTA or glutathione surfaces was not impaired in the presence of soluble glutathione or imidazole, respectively. The E26H retained its ability for dimerization and in accordance with this, the kinetic analysis also revealed no significant change of the catalytic constant upon E26H mutation.

Previous studies found that the Y7F mutation yields in the increase of the binding constant for glutathione while the activity for the CDNB substrate was dramatically reduced [23, 24]. The cause of this change is that the conserved Y7 active site residue plays a role in the catalytic mechanism. The modifications at the active site (like in case of Y7F mutation) can directly interfere with substrate binding and the enzymatic catalysis, but the E26 residue is located far from the active site, therefore, we have not expected change of the kinetic parameters. In accordance with this, the catalytic efficiencies of the wild‐type and mutant enzymes as well as their binding efficiencies to the glutathione affinity surface were almost identical. The results proved that recombinant proteins fused either to the wild‐type or to mutant *sj*GST can be detected based on the transferase activity of *sj*GST.

The affinity studies revealed that neither soluble glutathione nor imidazole interfere with the binding of *sj*GST(E26H) to Ni‐NTA or glutathione surface, respectively, indicating that the modified protein can be purified with both Ni‐NTA and glutathione affinity purification.

The in vitro comparison of wild‐type and modified *sj*GST was performed by using single *sj*GST molecules rather than using GST‐tagged fusion proteins, because the presence of a fusion partner was assumed to potentially interfere with ligand binding, enzyme activity and dimerization. But, we have tested the use of the E26H mutant *sj*GST for the purification of a fusion protein, as well. For this purpose we have chosen a human retroviral‐like protein ASPRV1 [42, 43, 44, 45, 46], especially its ASPRV1‐28 form. In order to prevent the self‐proteolysis of the recombinant protein, a mutation inactivating the aspartic protease domain was introduced (D212A). The fusion protein was purified first with Ni‐NTA, followed by glutathione affinity purification. Our results demonstrate efficient binding of the modified *sj*GST to both affinity surfaces, even in the presence of a fusion partner, the first purification step eliminated considerable amount of contaminants, and the second purification resulted in sufficient purity of GST‐tagged ASPRV1‐28.

Both polyhistidine (His_6_ or His_10_) and GST tags have their advantageous features from the viewpoint of the expression and purification of recombinant proteins which must be considered while choosing a proper fusion partner and a purification strategy [47]. The beneficial properties of these tags may be combined by (i) tagging the *N*‐ and *C*‐termini of the target protein with different tags (e.g., with polyhistidine and GST) [48], (ii) using a GST‐His [25] or a His‐GST dual tag (while using, e.g., pEX‐N‐His‐GST or pPB‐His‐GST bacterial expression vectors), or (iii) using the E26H mutant *sj*GST. The members of pGEX vector family can be used to express recombinant proteins that are fused to and *N*‐terminal *sj*GST tag. Although, plasmids coding for the E26H mutant *sj*GST are currently not available commercially, the E26H single point mutation can be easily introduced into the already existing expression constructs by using a simple PCR‐based site directed mutagenesis based on the protocols reported previously [29] or described in this paper. It is important to note that the ability of the E26H mutant *sj*GST for binding to Ni‐NTA surface may limit its application if is to be studied together with a His‐tagged protein (e.g., in pull‐down assay using Ni‐NTA surface). In such cases, the use of the wild‐type *sj*GST may be preferable. The E26H mutant is considered as a versatile variant of *sj*GST due to its improved ability for binding divalent ions, and due to the fact that its structural and enzymatic characteristics are identical with those of the wild‐type.

The recombinant proteins that are tagged with the E26H mutant *sj*GST might be potentially purified not only with the conventional glutathione affinity chromatography but also with IMAC. Ni‐NTA can be a good alternative of non‐affinity methods—for example, ion exchange or size‐exclusion chromatography—if the use of two purification steps is required for the purification of *sj*GST‐fused proteins. In this study we proved that the methods which have been established and optimized for the detection of the glutathione S‐transferase can be applied reliably in the case of the E26H mutant *sj*GST, because the structural and biochemical characteristics of the wild‐type protein remain unchanged upon the mutation.

## Author Contributions

**János András Mótyán:** conceptualization, methodology, software, formal analysis, supervision, funding acquisition, writing – original draft, visualization, data curation, investigation, validation. **Ágota Nagyné Veres:** conceptualization, methodology, formal analysis, investigation, data curation. **József Tőzsér:** conceptualization, validation, investigation, funding acquisition, writing – original draft, supervision, resources.

## Ethics Statement

The authors have nothing to report.

## Consent

The authors have nothing to report.

## Conflicts of Interest

The authors declare no conflicts of interest.

## Supporting information


**Figure S1:** Representative polyacrylamide gel images are shown as representatives of at least three parallel SDS‐PAGE analyses. The band intensities were determined by densitometry, the data are plotted in the graphs of Figure 3. The (a–e) gel images of Figure S1 belong to the (a–e) graphs of Figure 3.

## Data Availability

The data generated during this study are included in this published article (and its Supporting Information files). No datasets were analyzed during the current study.
