# Supplementary figures and images for: Characterization of the E26H Mutant Schistosoma japonicum Glutathione S‐Transferase
Source: Proteins. 2025 Jan 2;93(5):1054–66. doi: 10.1002/prot.26794 (PMC11968563; doi:10.1002/prot.26794)

**a**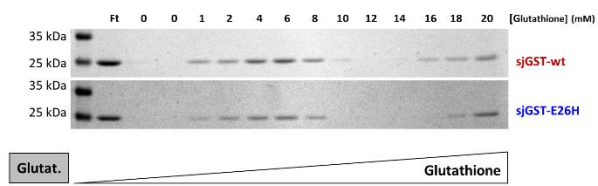**b**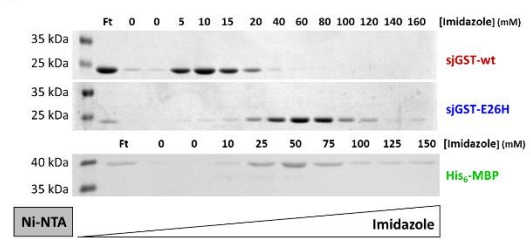**c**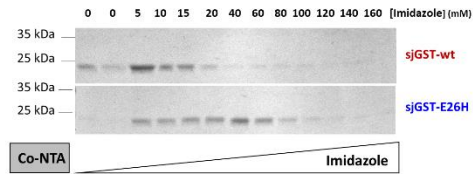**d**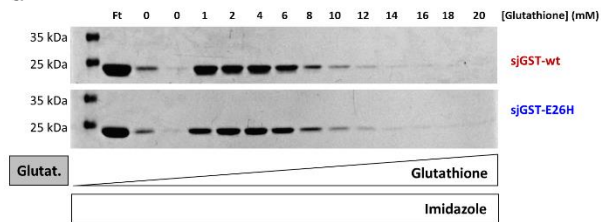**e**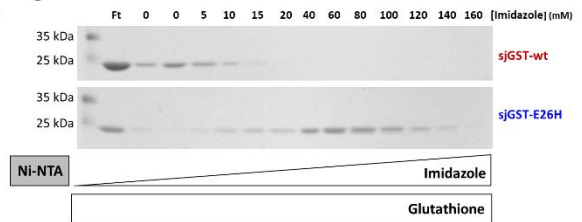

Supplement: Supplementary file 1 — Figure S1: Representative polyacrylamide gel images are shown as representatives of at least three parallel SDS‐PAGE analyses. The band intensities were determined by densitometry, the data are plotted in the graphs of Figure 3. The (a–e) gel images of Figure S1 belong to the (a–e) graphs of Figure 3. [file PROT-93-1054-s001.pdf]
